# Is pre-operative monocyte count-high-density lipoprotein ratio associated with postoperative acute kidney injury in isolated coronary artery bypass grafting?

**DOI:** 10.5830/CVJA-2022-055

**Published:** 2022-11-24

**Authors:** Hüseyin Şaşkın

**Affiliations:** Cardiovascular Surgery Clinic, Derince Training and Research Hospital, Health Sciences University, Kocaeli, Turkey

**Keywords:** coronary artery bypass grafting surgery, monocyteto-high-density lipoprotein cholesterol ratio, acute kidney injury

## Abstract

**Objective:**

Monocyte-to-high-density lipoprotein cholesterol ratio has emerged as an indicator of inflammation and oxidative stress in recent years. The aim of this study was to evaluate the association of monocyte-to-high-density lipoprotein ratio with postoperative acute kidney injury in isolated coronary artery bypass grafting.

**Methods:**

A total of 954 patients (672 males, mean age 60.8 ± 8.2 years), operated on between June 2014 and June 2022, at the same centre by the same team, for isolated coronary artery bypass grafting with cardiopulmonary bypass, whose preoperative serum creatinine level was < 1.5 mg/dl, were enrolled in the study. Patients were placed in group 1 if they had acute kidney injury in the early postoperative period (n = 161) and group 2 comprised those without (n = 793). Univariate and subsequent multivariate logistic regression analysis were done to determine significant clinical factors, and independent predictors of acute kidney injury.

**Results:**

Pre-operative monocyte count (p = 0.0001), monocyte count-high-density lipoprotein cholesterol ratio (p = 0.0001), C-reactive protein (p = 0.0001), erythrocyte sedimentation rate (p = 0.0001), mean platelet volume (p = 0.0001) and postoperative first- and third-day C-reactive protein levels (p = 0.0001) were significantly increased in group 1. Multivariate logistic regression analysis revealed that pre-operative elevated monocyte count (p = 0.0001), monocyte-high-density lipoprotein ratio (p = 0.0001), erythrocyte sedimentation rate (p = 0.0001), postoperative first-day C-reactive protein level (p = 0.0001), postoperative first-third day erythrocyte sedimentation rate (p = 0.002, p = 0.004, respectively) and mean platelet volume (p = 0.02, p = 0.0001, respectively) were independent predictors of early postoperative acute kidney injury in patients who had undergone isolated coronary artery bypass grafting.

**Conclusion:**

Pre-operative monocyte-high-density lipoprotein cholesterol ratio was found to be an independent predictor of acute kidney injury in the early postoperative period of isolated coronary artery bypass grafting.

Coronary artery bypass grafting (CABG) surgery is still the goldstandard treatment method for multi-vessel or left main coronary artery disease (CAD).^1^ With the developments in cardiopulmonary bypass (CPB) technology in recent years, these surgeries can be done with very low mortality and morbidity rates.^2^

As an important pathology, acute kidney injury (AKI) often follows cardiac surgery, resulting in extended intensive care unit (ICU) and hospital stays, and increased expenses and morbidity and mortality rates.^3^ AKI is a common complication after cardiac surgery, with an incidence of 19–42%, and renal replacement therapy may be required in 1–3% of these patients.^4^ The risk factors and pathophysiology of AKI following CABG have been described in the literature and have been the subject of many studies.^3^ The pathophysiology of AKI is complex and multifactorial; the injury site is typically the tubular cells, and toxins (exogenous and endogenous), inflammation, ischaemia– reperfusion injury, neurohormonal activation, metabolic factors and oxidative stress play a role in its aetiopathogenesis.^5^

Various biomarkers have been used recently to diagnose AKI, as one of the important causes of morbidity and mortality in the early postoperative period of CABG.^3^ Including white blood cell (WBC) count, leukocyte subtypes, platelets, mean platelet volume (MPV), C-reactive protein (CRP), neutrophil-to-lymphocyte ratio (NLR) and platelet-to-lymphocyte ratio (PLR), various inflammatory biomarkers have been shown to exist as important prognostic determinants in various cardiovascular diseases.^6^ Parlar et al. showed that increased NLR and PLR levels in the pre-operative and early postoperative periods were directly related to and independent biomarkers of the development of AKI in the early postoperative period.^7^

Monocytes are the main source of pro-inflammatory cytokines, and in atherosclerosis, modified low-density lipoprotein cholesterol (LDL-C) is removed by macrophages. These accumulate in the vessel wall and induce the release of inflammatory cytokines in the inflamed tissue, resulting in the production of inflammatory cholesterol ester-loaded plaque.^8^ High-density lipoprotein cholesterol (HDL-C), on the other hand, has anti-inflammatory, antioxidant and antiatherosclerotic effects by neutralising the pro-inflammatory and pro-oxidant effects of monocytes by inhibiting the migration of macrophages and LDL-C oxidation, in addition to cholesterol exit from these cells.^9^

The monocyte-HDL-C ratio (MHR) is a new and inexpensive marker of inflammation and can be easily obtained by calculations using blood cell analysis and lipid analysis.^10^ Recent studies have shown that MHR is used as a potential marker to predict mortality and major adverse cardiovascular events in patients with ST-elevation myocardial infarction (STEMI) who underwent primary percutaneous coronary intervention (pPCI).^11^

Kanbay et al. reported in their study that high MHR was associated with worse cardiovascular profile in predialytic chronic renal failure patients with decreased estimated glomerular filtration rate (eGFR) and that MHR was an independent predictor of major cardiovascular events during follow up.^12^

Again, Canpolat et al. reported that it was an independent predictor of recurrence of atrial fibrillation (AF) after atrialbased catheter ablation and was significantly associated with the presence of slow coronary flow.^13^ Lui et al. showed that high MHR levels were associated with poor functional outcomes in patients with acute ischaemic stroke.^14^ In this study we aimed to investigate the association of pre-operative MHR with early postoperative AKI in isolated CABG surgery.

## Methods

The medical records of a total of 1 404 patients who underwent isolated CABG between June 2014 and June 2022 were reviewed retrospectively. The operations were performed at the same centre by the same surgery team; 954 (67.9%) patients who had serum creatinine (sCr) levels of < 1.5 mg/dl who underwent isolated CABG with CPB were included in the study. The diagnosis of AKI was based on the highest sCr concentration measured during the first seven days after surgery compared with the baseline sCr concentration, defined as the last concentration measured before surgery.

Urine output was not used to define AKI, because it might have been altered by diuretics administered during anaesthesia and CPB.^7^ Patients who developed AKI in the early postoperative period were classified as group 1 (n = 161) and those with normal postoperative renal function were classified as group 2 (n = 793).

The diagnosis of AKI was made by comparing the baseline and postoperative sCr to determine the predefined significant change based on the kidney disease improving global outcomes (KDIGO) definition (increase in sCr by ≥ 0.3 mg/dl within 48 hours of surgery or increase in sCr to ≥ 1.5 times baseline within three days of cardiac surgery).^15^

We excluded patients previously diagnosed with end-stage renal disease who were on dialysis. Also excluded from the study were patients who had peripheral and carotid arterial disease, valvular heart disease, systemic inflammatory diseases, chronic obstructive pulmonary disease, congenital cardiac disease, malignancy, haematological proliferative diseases, autoimmune diseases, endocrinological disorders, immunosuppressive drug treatment within the past two weeks before surgery, advanced age (> 75 years), left ventricular systolic function disorder [left ventricular ejection fraction (LVEF) ≤ 30%], renal impairment (sCr ≥ 1.5 mg/dl), patients with low pre-operative haemoglobin levels (≤ 10 g/dl), the use of steroids or non-steroidal antiinflammatory drugs, the presence of signs of clinical infection [fever 37.5°C, CRP ≥ 5 mg/dl, erythrocyte sedimentation rate (ESR) > 20 mm/h or leukocyte count > 11 000 cells/μl] before surgery, patients who had an acute myocardial infarction and percutaneous coronary intervention in the last 30 days prior to operation, emergency operations, patients who required intra-aortic balloon pump, patients who were re-operated on due to haemodynamic instability or bleeding, patients who were operated on a beating heart or redo CABG. Additionally, patients for whom data such as sCr levels or urine output were missing and patients whose cardiac catheterisations were performed within the last 15 pre-operative days were excluded.

The demographic and clinical data of the patients were obtained from the hospital’s software system. Age, gender, smoking (defined as continuous or cumulative smoking for six or more months or at least six months every day; passive smoking refers to non-smokers inhaling the smoke from smokers’ breath for at least 15 minutes per day for more than one day per week), history of statin use, diabetes (including history of diabetes mellitus and newly diagnosed diabetes), hypertension (defined as history of hypertension and newly diagnosed hypertension), dyslipidaemia (defined as low HDL-C and high triglycerides; the cut-off values were selected at HDL-C < 40 mg/dl (1.04 mmol/l) and triglycerides ≥ 200 mg/dl (2.26 mmol/l) in both men and women), LVEF, laboratory parameters (haemoglobin, haematocrit, leukocyte count, thrombocyte count, monocyte count, LDL-C, HDL-C, total cholesterol, triglycerides, serum creatinine, urea and uric acid, eGFR, ESR and CRP), operation information, the number of grafts used, duration of CPB and aortic cross-clamp (ACC), use of blood products and length of stay in the ICU and hospital were recorded.

Laboratory examinations, including blood cell counts and lipid profiles, were routinely obtained within 24 hours of fasting upon admission to the hospital. Approximately 5–7 ml venous blood samples were placed pre-operatively into two types of sterile tubes: one with EDTA for blood count and one dry biochemistry tube for biochemical analysis.

Haematological parameters were calculated by an automated blood count device (Abbott CELL-DYN 3700; Abbott Laboratory, Abbott Park, Illinois, USA) following a waiting time of one hour. Serum levels of total cholesterol, LDL-C, HDL-C and triglycerides were determined in all specimens using an automatic multichannel biochemical analyser (Hitachi-7450, Hitachi, Tokyo, Japan) following routine laboratory procedures in our hospital. MHR was calculated by dividing the number of pre-operative monocyte counts by HDL-C level.^16^

This study complied with the Declaration of Helsinki and was carried out following approval of the Ethics Committee for Clinical Trials of Kocaeli Derince Training and Research Hospital of Health Sciences, University ethics committee approval number: 2022-106.

All patients were operated on with median sternotomy under general anaesthesia. Standard CPB was established with aortic and venous cannulations, systemic heparin (300 IU/kg) with maintenance of activated clotting time > 450 seconds. Hyperkalaemic cold blood cardioplegia was applied for cardiac arrest. Surgery was performed under moderate hypothermia (28–30oC). CPB flow was maintained at 2.2–2.5 l/min/m^2^, mean perfusion pressure between 50 and 80 mmHg, and haematocrit level between 20 and 25%. All distal anastomoses were done during the ACC period and proximal anastomoses onto the ascending aorta on a beating heart.

All patients were intubated and transferred postoperatively to the ICU. They were extubated following the onset of spontaneous breathing and normalisation of orientation and co-operation if the haemodynamic and respiratory functions were appropriate.

## Statistical analysis

Statistical analysis was performed using the SPSS software version 22.0 (SPSS Inc, Chicago, IL, USA). Among the data measured, those showing normal distribution are expressed as mean ± standard deviation; those not showing normal distribution are expressed as median (minimum–maximum). The data obtained by counting are given as percentages (%).

Among the data measured, the normality of distribution was evaluated by histogram or Kolmogorov–Smirnov test, the homogeneity of distribution was evaluated by Levene’s test for equality of variance. The difference between the groups was evaluated by the Student’s t-test in normal and homogenous distribution, and by the Mann–Whitney U-test in distribution that was not normal and homogenous. Among the data obtained by counting, the differences between the groups were evaluated by the parametric or non-parametric Pearson’s chi-squared test or Fisher’s exact test according to the distribution being parametric or not.

The effects of the risk factors suggested to be influential on the early postoperative AKI were studies through univariate logistic regression analysis. The multiple effects of the risk factors that were influential, or that were suggested to be influential in predicting the early postoperative AKI as a result of the univariate statistical analysis were studied through retrospective selective multivariate logistic regression analysis. The odds ratio, 95% confidence interval (CI) and the significance level for each of the risk factors were statistically significant at p < 0.05.

## Results

The demographic characteristics and clinical data of the patients are summarised in Table 1. There were no differences between the two groups in terms of demographics or clinical data. The pre-operative blood analysis and haematological parameters of the patients are summarised in Table 2. Monocyte counts (p = 0.0001), MHR (p = 0.0001), and ESR (p = 0.0001), MPV (p = 0.0001) and CRP levels (p = 0.0001) were significantly different between the groups.

**Table 1:** Demographic and clinical properties of the patients

	*Group I: AKI*	*Group II: non-AKI*	
*Patient characteristics*	*(n = 161)*	*(n = 793)*	*p-value*
Age, years (mean + SD)	60.3 + 8.4	60.9 + 8.2	0.48**
Male, n (%)	109 (67.8)	563 (71.0)	0.40*
Female, n (%)	52 (32.2)	230 (29.0)	
Hypertension, n (%)	71 (44.1)	325 (44.1)	0.47*
Diabetes mellitus, n (%)	52 (32.3)	267 (33.7)	0.74*
Smoking, n (%)	71 (44.1)	313 (38.5)	0.28*
Hyperlipidaemia, n (%)	67 (41.6)	367 (46.3)	0.27*
BMI (kg/m²) (mean + SD)	26.3 + 3.4	26.5 + 3.6	0.70**
Ejection fraction (%) (mean + SD)	53.8 + 8.5	54.4 + 8.7	0.40**
Basal heart rate (bpm) (mean + SD)	65.7 + 7.2	66.5 + 7.3	0.18**

AKI, acute kidney injury; BMI, body mass index.

*Pearson’s chi-squared test, **Student’s *t*-test

**Table 2:** Pre-operative blood results and haematological parameters of patients

*Parameters*	*Group I: AKI (n = 161) Mean + SD*	*Group II: non- AKI (n = 793) Mean + SD*	*p-value*
Haemoglobin (mg/dl)	13.4 + 1.5	13.6 + 1.6	0.26*
Creatinine (mg/dl)	0.73 + 0.28	0.74 + 0.29	0.77*
Urea (mg/dl)	40.7 + 4.2	40.6 + 3.9	0.78*
HbA, (%)	6.2 + 1.3	6.3 + 1.4	0.56*
eGFR (ml/min/1.73 r	96.2 + 31.4	95.4 + 30.6	0.69*
Leucocyte counts (10 ³ cells/ul)	8.2 + 1.6	8.2 + 1.7	0.79*
Thrombocyte counts (x10 cells/ul)	258 + 59	262 + 61	0.41*
Monocyte counts (x103 cells/ul)	0.79 + 0.15	0.63 + 0.17	0.0001*
CRP (mg/l)	0.93 + 0.65	0.67 + 0.47	0.0001*
ESR (mm/h)	9.93 + 4.79	8.04 + 3.90	0.0001*
MPV (fl)	9.02 + 1.03	8.62 + 0.82	0.0001*
HDL-C (mg/dl) (mmol/l)	38.6 + 6.7 1.00 + 0.17	38.4 + 7.1 0.99 + 0.18	0.75*
LDL-C(mg/dl) (mmol/l)	128.6 + 16.8 3.33 + 0.44	126.5 + 37.5 3.28 + 0.97	0.48*
Total cholesterol (mg/dl) (mmol/l)	191.1 + 43.8 4.95 + 1.13	187.8 + 41.1 4.86 + 1.06	0.36*
Triglycerides (mg/dl) (mmol/l)	156.8 + 69.6 1.77 + 0.79	158.9 + 73.4 1.80 + 0.83	0.74*
MHR	0.022 + 0.006	0.017 + 0.007	0.0001*

AKI, acute kidney injury; HDL-C, high-density lipoprotein cholesterol; eGFR, estimated glomerular filtration rate; LDL-C, low-density lipoprotein cholesterol; CRP, C-reactive protein; ESR: erythrocyte sedimentation rate; HbA1c, glycated haemoglobin; MPV, mean platelet volume; MHR, monocyte-to-highdensity lipoprotein cholesterol ratio.

*Student’s *t*-test.

The early postoperative blood analysis and haematological parameters of the patients are summarised in Table 3. Postoperative first- and third-day CRP levels (p = 0.0001), postoperative first- and third-day ESR (p = 0.0001) and MPV levels (p = 0.0001) were significantly different between the groups.

**Table 3:** Early postoperative blood resultsand haematological parameters of patients

	*Group I: AKI (n = 161)*	*Group II: non-AKI (n = 793)*	
*Characteristics*	*Mean + SD*	*Mean + SD*	*p-value*
Aortic cross-clamp time (min)	52.9 + 12.9	52.3 + 13.5	0.65*
Cardiopulmonary bypass time (min)	83.4 + 16.1	83.1 + 16.7	0.83*
Number of anastomoses	3.39 + 1.00	3.36 + 0.99	0.76*
Amount of drainage (ml)	361 + 140	350 + 129	0.34*
Intubation time (h)	5.76 + 1.41	5.62 + 1.48	0.25*
Stay in the intensive care unit (h)	22.81 + 3.57	21.00 + 2.19	0.0001*
Total duration of hospital stay (days)	8.96 + 3.14	6.06 + 1.61	0.0001*
Use of blood products, n (%)	57 (35.4)	261 (32.9)	0.54**
Use of inotropic support, n (%)	13 (8.1)	53 (6.7)	0.53**

AKI, acute kidney injury; CRP, C-reactive protein; ESR, erythrocyte sedimentation rate; MPV, mean platelet volume.

*Student’s t test.

The intra-operative and postoperative data of the patients are shown in Table 4. Intubation time (p = 0.0001), stay in the ICU (p = 0.0001) and length of hospital stay (p = 0.0001) were significantly different between the groups.

**Table 4:** Intra-operative and postoperative data of the patients

	*Group I: AKI (n = 161)*	*Group II: non-AKI (n = 793)*	
*Characteristics*	*Mean + SD*	*Mean + SD*	*p-value*
Aortic cross-clamp time (min)	52.9 + 12.9	52.3 + 13.5	0.65*
Cardiopulmonary bypass time (min)	83.4 + 16.1	83.1 + 16.7	0.83*
Number of anastomoses	3.39 + 1.00	3.36 + 0.99	0.76*
Amount of drainage (ml)	361 + 140	350 + 129	0.34*
Intubation time (h)	5.76 + 1.41	5.62 + 1.48	0.25*
Stay in the intensive care unit (h)	22.81 + 3.57	21.00 + 2.19	0.0001*
Total duration of hospital stay (days)	8.96 + 3.14	6.06 + 1.61	0.0001*
Use of blood products, n (%)	57 (35.4)	261 (32.9)	0.54**
Use of inotropic support, n (%)	13 (8.1)	53 (6.7)	0.53**

*Student’s *t*-test, **Pearson’s chi-squared test.

According to the KDIGO classification, 59.6% (n = 96) of the patients were stage I, 36.1% (n = 56) were stage II and 4.3% (n = 7) were stage III AKI. Renal failure requiring dialysis developed in three patients with stage III AKI.

Mortality in the first postoperative month occurred in six patients (3.7%) in group 1 and in seven patients (0.9%) in group 2. The difference between the groups was statistically highly significant (p = 0.005).

The results of univariate and multivariate regression analysis of patients who developed AKI in the early postoperative period are shown in Tables 5 and 6. In the multivariate analysis, the variables that were found to be statistically significantly associated with postoperative AKI in univariate analysis, were increased pre-operative monocyte counts (p = 0.0001), MHR (p = 0.0001) and pre-operative ESR levels (p = 0.001); increased postoperative first-day CRP levels (p = 0.0001), postoperative first- and third-day ESR levels (p = 0.002, p = 0.004, respectively) and MPV levels (p = 0.02, p = 0.0001, respectively). These were found to be independent predictors of early postoperative AKI.

**Table 5:** Univariate and multivariate regression analysis of pre-operative risk factors for postoperative acute kidney injury

	*Postoperative AKI*				
*Risk factors*	*Unadjusted OR (95% CI)*	*p*	*R2 square*	*Adjusted OR (95% CI)*	*p*
Haemoglobin	1.07 (0.95-1.20)	0.26	-	-	-
Creatinine	0.92 (0.51-1.66)	0.77	-	-	-
Urea	1.02 (0.96-1.05)	0.78	-	-	-
HbA 1c	0.97 (0.86-1.09)	0.58	-	-	-
eGFR	0.96 (0.92-1.00)	0.34	-	-	-
Leucocyte count	0.91 (0.87-0.95)	0.79	-	-	-
Thrombocyte count	0.99 (0.96-1.02)	0.41	-	-	-
Monocyte count	1.20 (1.12-1.28)	0.0001	0.21	1.39 (0.96-2.01)	0.0001
CRP	1.90 (1.47-2.45)	0.0001	0.29	1.26 (0.82-1.95)	0.29
ESR	1.12 (1.07-1.16)	0.0001	0.30	1.11 (1.05-1.17)	0.0001
MPV	1.13 (1.35-1.96)	0.0001	0.17	1.12 (0.85-1.48)	0.43
HDL-C	1.01 (0.98-1.04)	0.75	-	-	-
LDL-C	0.99 (0.97-1.01)	0.50	-	-	-
Total cholesterol	0.99 (0.98-1.00)	0.36	-	-	-
Triglycerides	1.00 (0.99-1.01)	0.74	-	-	-
MHR	25.77 (22.37-29.17)	0.0001	0.19	21.57 (13.09-34.54)	0.0001

AKI, acute kidney injury; HDL-C, high-density lipoprotein cholesterol; eGFR, estimated glomerular filtration rate; LDL-C, low-density lipoprotein cholesterol;

CRP: C-reactive protein; ESR: erythrocyte sedimentation rate; HbA1c, glycated haemoglobin; MPV, mean platelet volume; MHR, monocyte-to-high-density

lipoprotein cholesterol ratio.

**Table 6:** Univariate and multivariate regression analysis of peri-operative and postoperative risk factors for postoperative acute kidney injury.

	*Postoperative AKI*				
*Risk factors*	*Unadjusted OR (95% CI)*	*p-value*	*R2*	*Adjusted OR (95% CI)*	p-value
Postoperative first-day haemoglobin	1.11 (0.96-1.29)	0.17	-	-	-
Postoperative third-day haemoglobin	1.04 (0.98-1.10)	0.16	-	-	-
Postoperative first-day leukocytes	0.97 (0.91-1.03)	0.24	-	-	-
Postoperative third-day leukocytes	0.94 (0.89-0.99)	0.32	-	-	-
Postoperative first-day CRP	1.12 (1.09-1.15)	0.0001	0.13	1.08 (1.05-1.11)	0.0001
Postoperative third-day CRP	1.09 (1.05-1.13)	0.0001	0.09	1.05 (0.99-1.11)	0.08
Postoperative first-day ESR	1.03 (1.01-1.05)	0.0001	0.10	1.02 (1.01-1.03)	0.002
Postoperative third-day ESR	1.05 (1.02-1.08)	0.0001	0.12	1.04 (1.01-1.07)	0.004
Postoperative first-day MPV	2.03 (1.67-2.46)	0.0001	0.16	1.45 (1.07-1.96)	0.02
Postoperative third-day MPV	1.99 (1.60-2.49)	0.0001	0.12	1.82 (1.36-2.44)	0.0001
Aortic cross-clamp time	1.01 (0.99-1.03)	0.65	-	-	-
Number of anastomoses	1.03 (0.87-1.22)	0.75	-	-	-
CPB time	1.02 (0.99-1.05)	0.84	-	-	-
Intubation time	1.07 (0.95-1.20)	0.25	-	-	-
Use of blood products	0.90 (0.63-1.23)	0.54	-	-	-
Use of inotropic support	0.82 (0.44-1.54)	0.53	-	-	-
Amount of drainage	1.00 (0.99-1.01)	0.34	-	-	-

CPB, cardiopulmonary bypass; CRP, C-reactive protein; ESR, erythrocyte sedimentation rate; AKI, acute kidney injury.

**Fig. 1:**
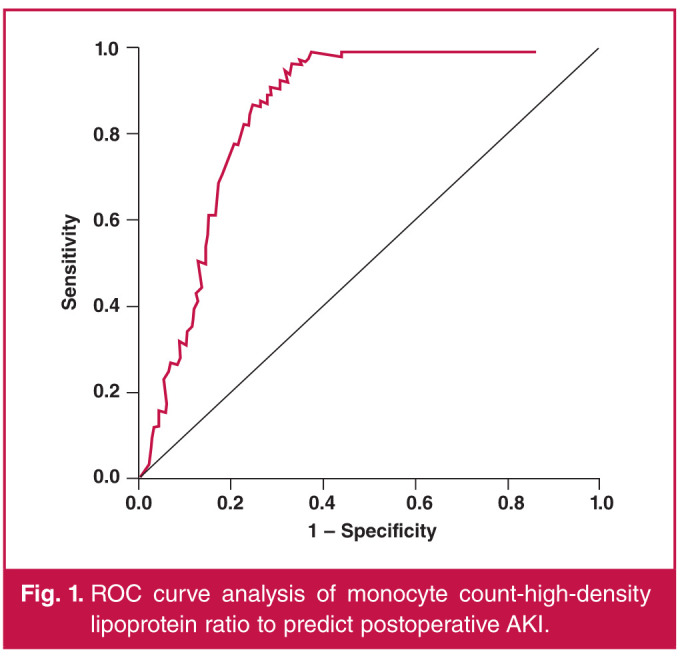
ROC curve analysis of monocyte count-high-density lipoprotein ratio to predict postoperative AKI.

The ROC curves for MHR were associated with postoperative AKI following isolated CABG (Fig. 1). The area under the curve for MHR was 0.851 (95% CI: 0.826– 0.876; p = 0.0001). Using a cut-off value of 0.0212, MHR predicted postoperative AKI with a sensitivity of 78.9% and a specificity of 78.4%.

## Discussion

In this study, elevated pre-operative MHR was associated with early postoperative AKI after isolated CABG operation. To the best of our knowledge, this is one of the first studies to evaluate the relationship between MHR and AKI after CABG operation.

One of the most important treatment modalities preferred for many patients with coronary artery disease is CABG, and patients undergoing this surgery are at risk of serious complications.^17^ One of these complications is AKI, with an incidence rate of 5–42%, and which is associated with higher patient morbidity and mortality rates, length of hospital stay and cost.^18^

AKI, which has been the subject of intense research in the last 20 years, is the sudden loss of kidney function characterised by an acute increase in sCr concentration.^19^ There are multiple diagnostic criteria for AKI developing after cardiac surgery. Although there are many new studies available for the diagnosis of advanced AKI, sCr and blood urea nitrogen (BUN) used together with urine output remain the cornerstone of diagnosis in clinical practice.^20^ In our study, we used sCr and BUN values for the diagnosis of AKI.

While AKI causes an increase of 10–30% in hospital mortality rate, this increase reaches up to 40–60% when dialysis is required.^21^ Hobson et al. reported that 43% of their patients developed AKI during hospital follow up after cardiac surgery.^22^ For standardisation, patients operated on for only isolated CABG using standard CPB were enrolled in our study. Postoperative AKI and acute renal failure occurred in 16.9 and 4.3% of the patients, respectively, in parallel with the literature.

The incidence of AKI following cardiac surgery depends on its definition. The risk, injury, failure, loss of kidney function, and end-stage kidney disease (RIFLE) classification, acute kidney injury network (AKIN) criteria and kidney disease improving global outcomes (KDIGO) stages, are all practical predictors of AKI after cardiac surgery.^15^ In our study, AKI was defined according to the KDIGO criteria.

The pathophysiology of AKI developing after cardiac surgery is complex and multifactorial, including renal ischaemia– reperfusion injury, exogenous and endogenous toxins, radiocontrast agent use, neurohormonal activation, metabolic factors, hypoproteinaemia, inflammation and oxidative stress.^23^ Ischaemia–reperfusion is the most common cause of post-cardiac surgery AKI and is associated with the pathological features of acute tubular necrosis.^24^ In addition, there are studies stating that inflammation plays a key role in the initiation and progression of AKI that develops after cardiac surgery, while oxidative stress and haemolysis are pathways that complement inflammation.^25^ In general, it has been shown that direct or indirect suppression of the inflammatory response could significantly reduce the degree of kidney injury in an animal model, which manifested as a relative decrease in sCr levels and a reduction in tubular necrosis.^26^

Various risk models have been defined to predict CABG-related mortality and morbidity, and many blood markers have been investigated in this regard.^27^ In recent years, novel structural biomarkers used in clinical practice as early and rapid indicators of AKI developing after cardiac surgery have facilitated evaluation of disease occurrence and progression.^28^ A number of urine and blood biomarkers, including neutrophil gelatinase-associated lipocalin (NGAL), interleukin-18, cystatin C and kidney injury molecule-1 (KIM-1) are elevated before sCr levels and facilitate the early diagnosis of AKI.^29^ Biomarkers such as urinary liver fatty acid binding protein (L-FABP), urinary NGAL, serum L-FABP, heart type FABP, KIM-1 and interleukin-18 have been found statistically significantly higher in patients who develop AKI after cardiac surgery.^28^

Novel biomarkers are required to replace current clinical risk classifications in order for clinicians to design new clinical trials to take appropriate preventative measures for AKI and evaluate clinical properties to determine effective treatments.^29^ In our study, we aimed to identify possible pre-operative blood biomarkers as candidates that would improve the prediction of AKI, alone or in combination with the existing clinical scoring tools, in patients undergoing isolated CABG with CPB.

In recent studies, it has been shown that various inflammatory biomarkers, including WBC, leukocyte subtypes, platelet count, CRP level, NLR and PLR are important prognostic determinants in various cardiovascular diseases.^7^ Meta-analysis studies by Wu et al. reported that strong haematological inflammatory indicators such as haematocrit and red blood cell distribution width were associated with contrast-induced nephropathy.^30^ In another study, Parlar et al. reported that NLR values measured in the first four postoperative days of CABG were useful in predicting AKI during hospital stay.^31^ In our study, we found that CRP, ESR and MPV values, which are important inflammatory biomarkers, were significantly higher in patients who developed AKI both pre- and early postoperatively.

Neutrophils, lymphocytes, monocytes and eosinophils, which are leukocyte subtypes, have separate roles in inflammation, host defence and tissue repair.^32^ Monocytes, which are innate and one of the basic components of the human immune system, are circulating leukocytes that play an important role in inflammation and tissue remodelling.^25^ Activated macrophages and monocytes secrete various pro-inflammatory and pro-oxidant mediators, attaching to the inner surface of the arterial wall and initiating the atherosclerotic process.^32^ Monocyte counts have recently been suggested as a predictor of coronary events, and increased monocyte counts have been associated with adverse cardiovascular endpoints in coronary artery patients.^9^ In our study, similar to other studies, we found that pre-operative high monocyte count was an independent predictor of postoperative AKI development.

HDL-C, a component of the lipid profile, can prevent free cholesterol and triglyceride accumulation in the vessels; it protects endothelial cells against the negative effects of LDL-C by preventing its oxidation.^33^ Studies have reported that HDL-C has pleiotropic protective functions including antiinfectious, antithrombotic, anti-inflammatory, antioxidant and immunomodulatory properties.^14^ In addition, HDL-C inhibits the endothelial expression of adhesion molecules and prevents the infiltration of monocyte cells into the arterial wall.^27^

The inverse relationship between HDL-C levels and cardiovascular disease risk has been well known for a long time.^33^ Many prospective studies from different racial and ethnic groups around the world have confirmed that HDL-C is a strong, consistent and independent predictor of cardiovascular events (myocardial infarction, ischaemic stroke).34 The protective effects of high HDL-C levels on the risk of myocardial infarction have been demonstrated in numerous epidemiological studies.^35^

Zhang et al. reported in their community-based study on a hypertensive Chinese population that high HDL-C values were an important protective factor in ischaemic stroke.^36^ Canpolat et al. also examined HDL-C level in the formation of AF due to its anti-inflammatory and antioxidant effects, and it was shown that low HDL-C values were significantly associated with AF.^13^ In our study, there was no significant difference between the groups in terms of HDL-C values.

It has been suggested that MHR has an important role in systemic inflammation and may be a possible predictor of the development and progression of atherosclerosis.^36^ In recent studies, it has been reported that MHR is associated with cardiovascular events in chronic kidney disease.^37^ In another study, Cetin et al. emphasised that increased MHR, a new marker of inflammation, is an independent predictor of many major cardiovascular adverse events, including stent thrombosis and mortality after pPCI in STEMI patients.^38^ In another study, it was reported that MHR may be a better parameter than NLR and CRP in estimating the severity of CAD in STEMI patients treated with pPCI.^39^

Considering the large number of studies reporting the relationship between high monocyte counts and low HDL-C levels in inflammatory disorders, in our study, we focused on understanding whether MHR is appropriate for early diagnosis and prediction of the progression of AKI developing after CABG, in which ischaemia–reperfusion injury and inflammation play a major role. As a result, we included 954 patients who underwent isolated CABG with CPB to investigate the relationship between pre-operative MHR and early postoperative AKI in our study, and we found that increased MHR was an independent predictor of early postoperative AKI after adjusting for confounding factors. Therefore, MHR can be used as a valuable and costeffective predictor of clinical outcomes in patients undergoing isolated CABG with CPB.

## Limitations

A few limitations to our study should be mentioned. The biggest and primary limitation was that it included a limited, retrospective study population prone to prejudice, and was a single-centre study unlike multi-centre and cross-sectional studies. Second, due to the lack of urine output values, only sCr values were used to determine whether a patient met the criteria for AKI. Third, it was not very practical to determine the presence of AKI as it was not possible to fully monitor the urine output, since the urinary catheters inserted were usually removed from the patients approximately two to three days after the surgery. Fourth, we could not observe time-dependent changes in MHR values and postoperative AKI grade due to the retrospective study design. Therefore, we were unable to evaluate causal relationships between MHR and the development and/or progression of postoperative AKI. Fifth, the inflammatory process has dynamics and persistence, but MHR was not dynamically measured multiple times in our study. Therefore, the dynamic trend and value of the indicator were not reflected in this study.

## Conclusion

We believe that MHR, which can easily be obtained from a simple complete blood count and is an inexpensive inflammatory marker, may be helpful in predicting the development of early postoperative AKI in patients who undergo isolated CABG with CPB. Although we were not able to establish a causal relationship in this study, some clinical implications may be obtained if the results of this study are confirmed by large-scale prospective studies.
